# Supplementary material for: Time‑resolved multi-omic analysis of paclitaxel exposure in human iPSC‑derived sensory neurons unveils mechanisms of chemotherapy‑induced peripheral neuropathy
Source: Cell Death Dis. 2026 Feb 10;17(1):211. doi: 10.1038/s41419-026-08445-2 (PMC12921266; doi:10.1038/s41419-026-08445-2)
Supplement: Supplementary file 15 — Dataset 1. [file 41419_2026_8445_MOESM15_ESM.zip › CDD_Open_Data/04_RNA_Seq/Timeline_panel_plots_etc_2023-01-23_1609.zip!/Panel_Plots_etc/Evidence_Plots/72h/evidence_plot_standard contrasts_msigdb_go_bp_72h_PTX_vs_DMSO_ID0_M23579_GOBP_NEGATIVE_REGULATION_OF_MACROPHAGE_ACTIVATION.pdf]

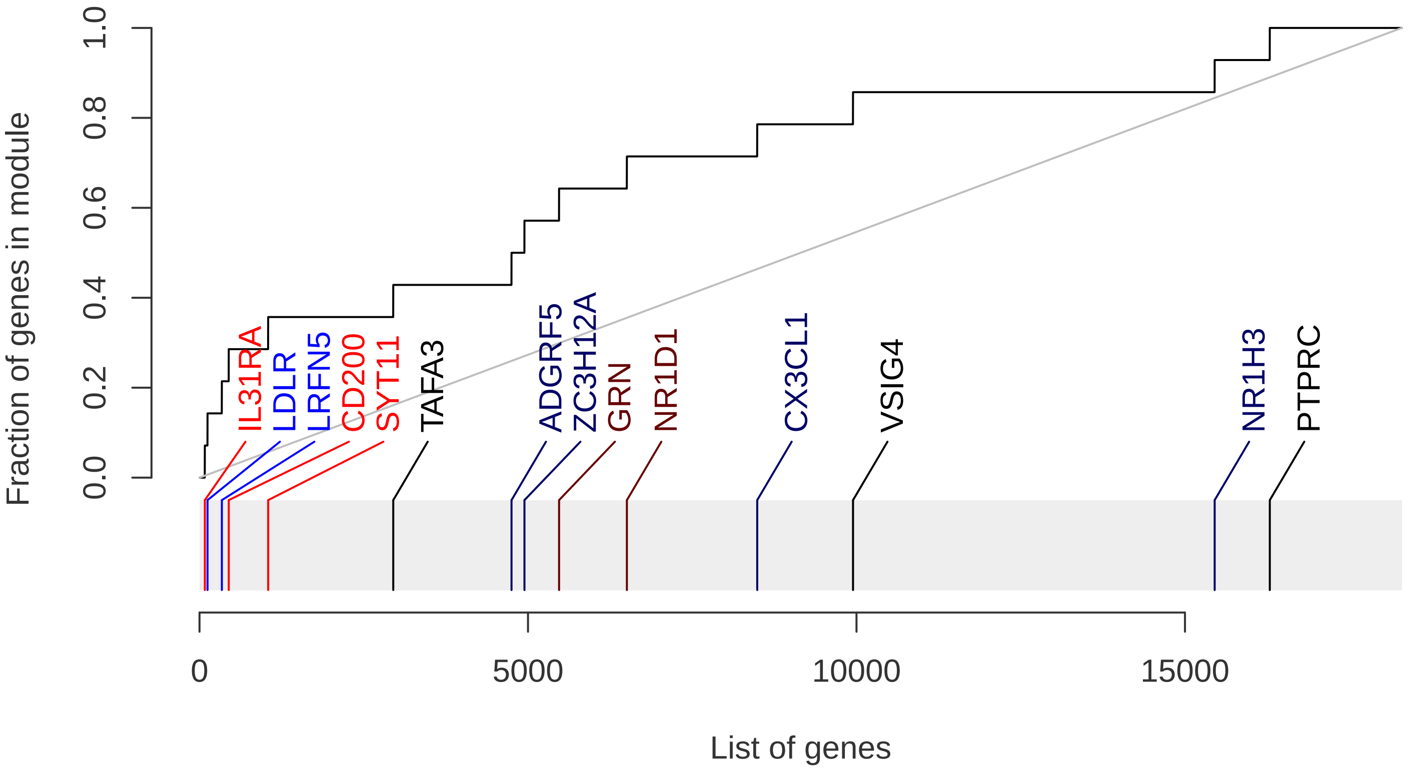

Fraction of genes in module
0.0
0.2
0.4
0.6
0.8
1.0
IL31RA
LDLR
LRFN5
CD200
SYT11
TAFA3
ADGRF5
ZC3H12A
GRN
NR1D1
CX3CL1
VSIG4
NR1H3
PTPRC
0
5000
10000
15000
List of genes
